# High Prevalence of Hepatitis Delta Virus among Persons Who Inject Drugs, Vietnam

**DOI:** 10.3201/eid2103.141147

**Published:** 2015-03

**Authors:** Naomi Hall, Linh Nguyen Thuy, Trinh Do Thi Diem, Allison Waters, Linda Dunford, Jeff Connell, Michael Carr, William Hall, Lan Anh Nguyen Thi

**Affiliations:** National Virus Reference Laboratory, University College Dublin, Dublin, Ireland (N. Hall, A. Waters, L. Dunford, J. Connell, M. Carr, W. Hall);; Laboratory for Molecular Diagnostics, National Institute of Hygiene and Epidemiology, Ha Noi, Vietnam (L.N. Thuy, T.D.T. Diem, L.A.N. Thi)

**Keywords:** Hepatitis delta virus, hepatitis, viruses, HDV, Vietnam, serology, phylogenetics, viral loads, persons who inject drugs

**To the Editor:** Hepatitis delta virus (HDV) is a small RNA virus that infects and persists only in persons whose samples test positive for hepatitis B surface antigen (HBsAg) (*1*). Phylogenetic analysis has revealed 8 HDV genotypes (*2*) with evidence of distinct global geographic distributions and pathogenicity (*3*,*4*). The implications of HDV infection in Vietnam have been unclear. Studies of persons who have chronic illness caused by HBV in populations of southern and northern Vietnam reported no cases or low prevalence (1.3%), respectively (*5*,*6*). In contrast, our multicenter study of chronically HBV-infected persons in 2009 showed a higher overall HDV seroprevalence rate of 10.7% (34/318) (*7*). These rates varied among regions of Vietnam and groups that had varying risk factors for infection. Higher rates were observed among persons who inject drugs (PWIDs) (20/78, 25.6%), commercial sex workers (5/57, 8.8%), and military recruits (8/45, 17.8%). A 2013 study, in which PCR-based methods were used, reported a high rate of HDV RNA detection (41/266 ,15.4%) in a cohort of HBV-infected persons in the city of Ha Noi (also known as Hanoi) collected during 2000–2009 (*8*). Illnesses of these patients ranged from acute hepatitis to severe liver disease, but injection of drugs was not reported. To better clarify the prevalence of HDV, we conducted serologic and molecular testing focusing on PWIDs from different geographic regions of Vietnam.

During 2010–2011, we screened consecutive samples (n = 1,999) from PWIDs from 5 centers (Ha Noi and Hai Phong in northern, Da Nang and Khanh Hoa in central, and Can Tho in southern Vietnam) for HBsAg. In each center, we recruited PWIDs to obtain 200 participants per year following national guidelines for annual sentinel surveillance of HIV (http://www.vaac.gov.vn/Download.aspx/C64DBE4BB9074A489283056ACF639780/1/Huong_dan_giam_sat_trong_diem_2010.doc). Ethical approval for the study was obtained from the National Institute of Hygiene and Epidemiology in Ha Noi. Samples collected from 300 (15%) persons were HBsAg positive, consistent with our previous study (*7*). Of these, 294 were subsequently screened by using ELISA for anti-HDV IgG; reactive samples were tested for HDV IgM. HDV IgG was detected in 45/294 (15.3%) samples; 20 were also HDV IgM positive (6.8% total; 44.4% of IgG-positive samples). Serologic analysis revealed considerable differences in prevalence by geographic region. HDV seroprevalence rates were high among PWIDs from northern Vietnam (30.2% and 29.4% in Ha Noi and Hai Phong, respectively), but a lower seroprevalence rate was observed in Da Nang (5.3%), and intermediate rates were found in Khanh Hoa (8.1%) and Can Tho (12.5%) in southern Vietnam (Technical Appendix Tables 1, 2).

We analyzed anti-HDV–positive samples (n = 41) for the presence of HDV RNA using a quantitative real-time PCR. HDV RNA was detected in 25/41 (61%) of IgG-seropositive samples (median 1.2 × 10^4^ copies/mL, range 0–1.8 × 10^7^ copies/mL) and 19/19 (100%) of IgM-seropositive samples (median 1.2 × 10^6^ copies/mL, range 4.3 × 10^2^–1.7 × 10^7^ copies/mL). The viral loads of HDV IgM-positive samples were significantly higher than those of IgM-negative samples (p<0.0001) (Technical Appendix Figure 1); however, when only samples with detectable HDV RNA from the IgM negative and positive groups were analyzed, there was no statistically significant difference in viral titer (p = 0.45; Technical Appendix Figure 2). Comparison of HDV RNA and HDV IgM seroresponses showed evidence of superinfection with HDV persistence in 6 cases (HDV IgM negative/RNA positive; 6/22, 27.3%; Technical Appendix Figure 1). The 6 samples that were IgM negative for detectable RNA (median 2.9 × 10^5^ copies/mL, range 1.1 × 10^3^–1.8 × 10^7^ copies/mL) highlight the limitation of using IgM as a surrogate marker for HDV replication; therefore, HDV RNA investigation is more appropriate for IgG-positive samples.

To identify the genotypes of HDV involved, we completed nucleotide sequencing and phylogenetic analysis of HDV from 17 viremic patient samples from Ha Noi, Hai Phong, Da Nang, Khanh Hoa, and Can Tho collected from another study cohort during 2008–2011 (Figure) (*7*). Most (12/17, 71%) samples were HDV genotype 1 from both northern and southern Vietnam; 5 (29%) HDV genotype 2 species were identified in 4 samples from Hai Phong in northern and 1 sample from Da Nang in central Vietnam. The finding that HDV-1 was the predominant genotype is consistent with reports by Sy et al. (19/21 HDV-1; 2/21 HDV-2) (*8*), suggesting that HDV-1 is the predominant genotype in all parts of the country.

**Figure:**
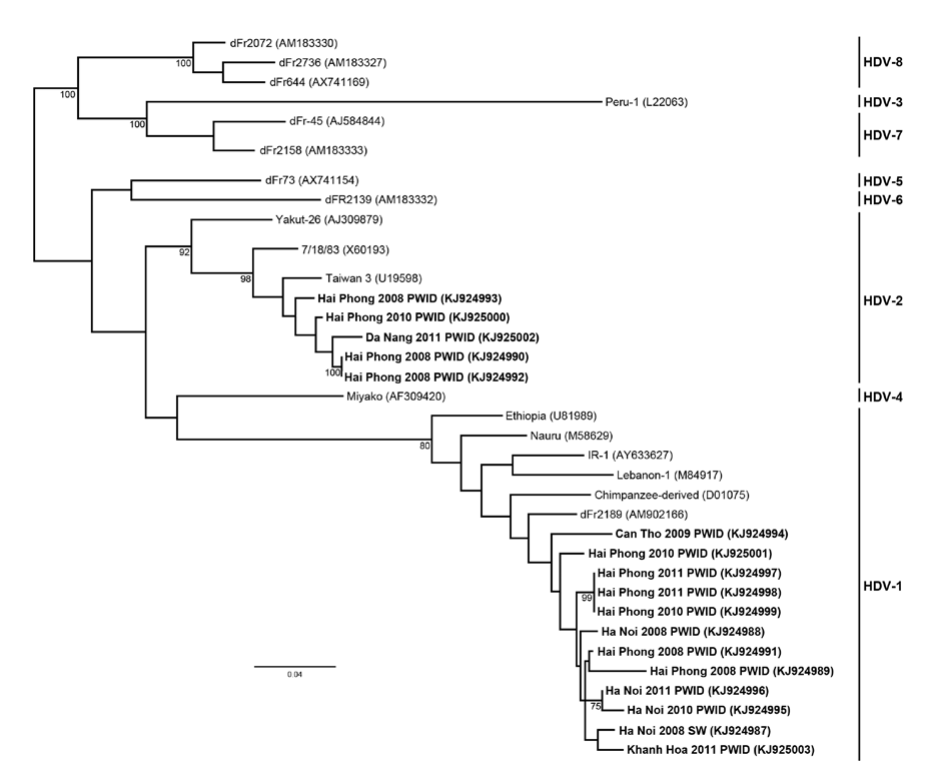
Maximum-likelihood phylogenetic tree of hepatitis delta virus (HDV) genotypes 1 and 2 from Vietnam. A 472-nt fragment (corresponding to nucleotides 802–1,273 from HDV isolate C15; GenBank accession no. KF660600) was used to construct the phylogram. HDV genotyping was done by using amplification and bidirectional sequencing of the *R*_0_ region as described by Le Gal et al. (*2*). Bootstrap resampling was done for 1,000 replicates of the dataset using the neighbor-joining algorithm; values >70% are shown at the nodes. Bold text indicates samples from patients in Vietnam; location, year, and risk group are indicated. Genbank accession numbers are shown in parentheses. Scale indicates substitutions per position. PWID, person who injects drugs; SW, sex worker.

This study, the previous report from the NIHE laboratory (*7*), and data from Sy et al. (*8*,*9*) indicate that HDV is highly prevalent in Vietnam, particularly in the northern part of the country, contrary to previous reports (*5*,*6*,*10*). In particular, our findings indicate that increased efforts are needed to improve HBV vaccination rates among PWIDs and others with risk factors for infection. Over time, these interventions may help reduce the effects of hepatitis virus–related liver disease. We also intend to study HDV in other high-risk groups, including commercial sex workers and men who have sex with men.

Technical Appendix Tabular summary of frequency of cases of hepatitis delta virus among persons who inject drugs and statistical analysis of data in study regions of Vietnam.
