# Supplementary material for: High Prevalence of Hepatitis Delta Virus among Persons Who Inject Drugs, Vietnam
Source: Emerg Infect Dis. 2015 Mar;21(3):540–3. doi: 10.3201/eid2103.141147 (PMC4344271; doi:10.3201/eid2103.141147)
Supplement: Technical Appendix — Tabular summary of frequency of cases of hepatitis delta virus among persons who inject drugs and statistical analysis of data in study regions of Vietnam. [file 14-1147-Techapp-s1.pdf]

Article DOI: http://dx.doi.org/10.3201/eid2103.141147

High Prevalence of Hepatitis Delta Virus among Persons Who Inject Drugs, Vietnam

Technical Appendix

Tabular summary of frequency of cases of hepatitis delta virus among persons who inject drugs and statistical analysis of data in study regions of Vietnam

Technical Appendix Table 1. Hepatitis B surface antigen seropositivity in persons who inject drugs from 5 study regions in Vietnam*

	Northern no. (%)		Central no. (%)	Southern no. (%)		
Group/HBV Serostatus	Ha Noi	Hai Phong	Da Nang	Khanh Hoa	Can Tho	Total no. (%)
PWIDs	400	400	400	400	399	1,999
HBsAg (+)	43 (10.8)	51 (12.8)	76 (19)	74 (18.5)	56 (14)	300 (15)

*HBV, hepatitis B virus; PWIDs, persons who inject drugs; HBsAg, hepatitis B surface antigen.

Technical Appendix Table 2. Hepatitis delta virus IgG and IgM seropositivity in hepatitis B surface antigen-positive persons who inject drugs in 5 study regions in Vietnam*

Characteristics	Northern, no. (%)		Central, no. (%)	Southern, no. (%)		Total, no. (%)
Serologic Markers	Ha Noi,. 43	Hai Phong, 51	Da Nang, 76	Khanh Hoa, 74	Can Tho, 56	294*
HDV IgG (+)	13 (30.2)	15 (29.4)	4 (5.3)	6 (8.1)	7 (12.5)	45 (15.3)
HDV IgM (+)	5 (11.6)	8 (15.6)	2 (2.6)	5 (6.7)	0 (0)	20 (6.8)

*Of the 300 hepatitis B surface antigen–positive samples (Table 1), 294 were available for analysis; the remaining 6 had insufficient sample volumes. HDV, hepatitis delta virus.

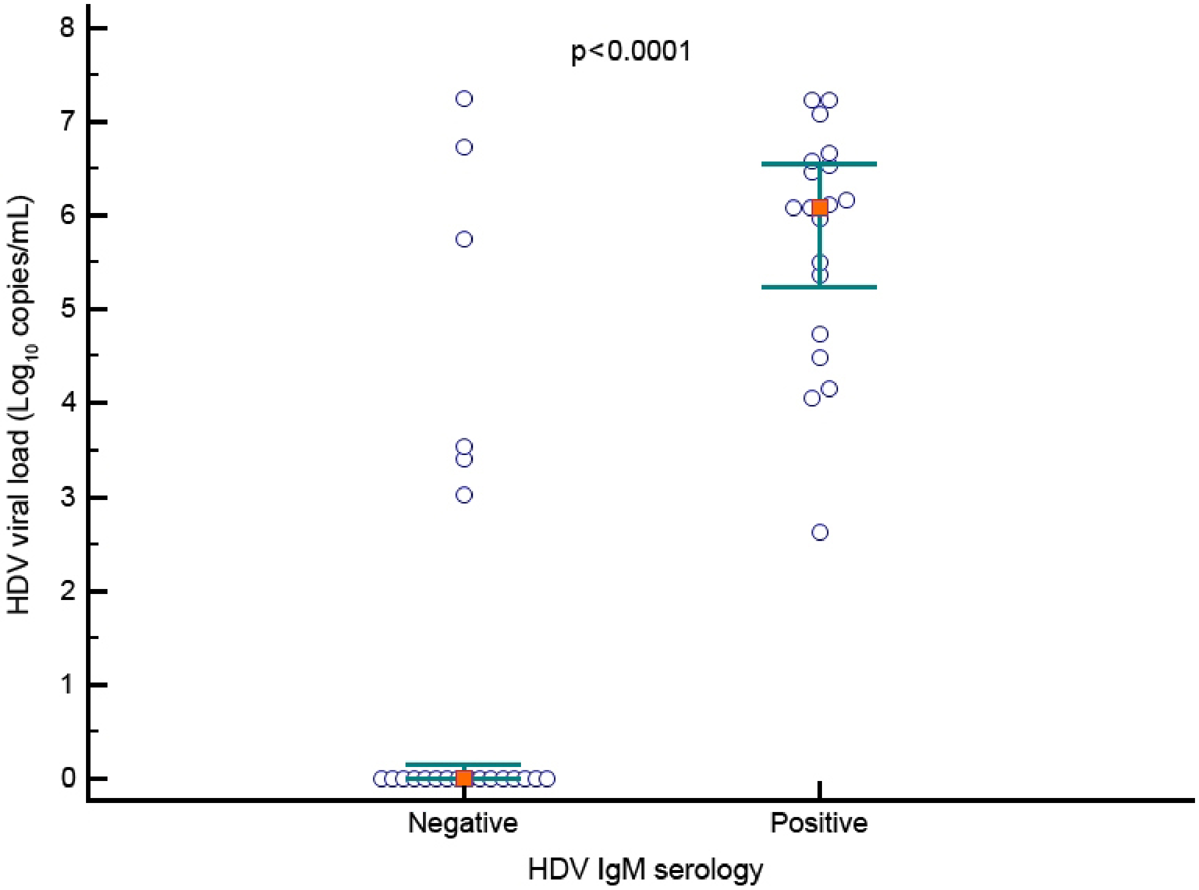

Technical Appendix Figure 1. HDV RNA log_{10} copies/mL in IgM-negative (n=22) and IgM-positive (n=19) samples. The orange square indicates the median and the error bars indicate 95% CI.

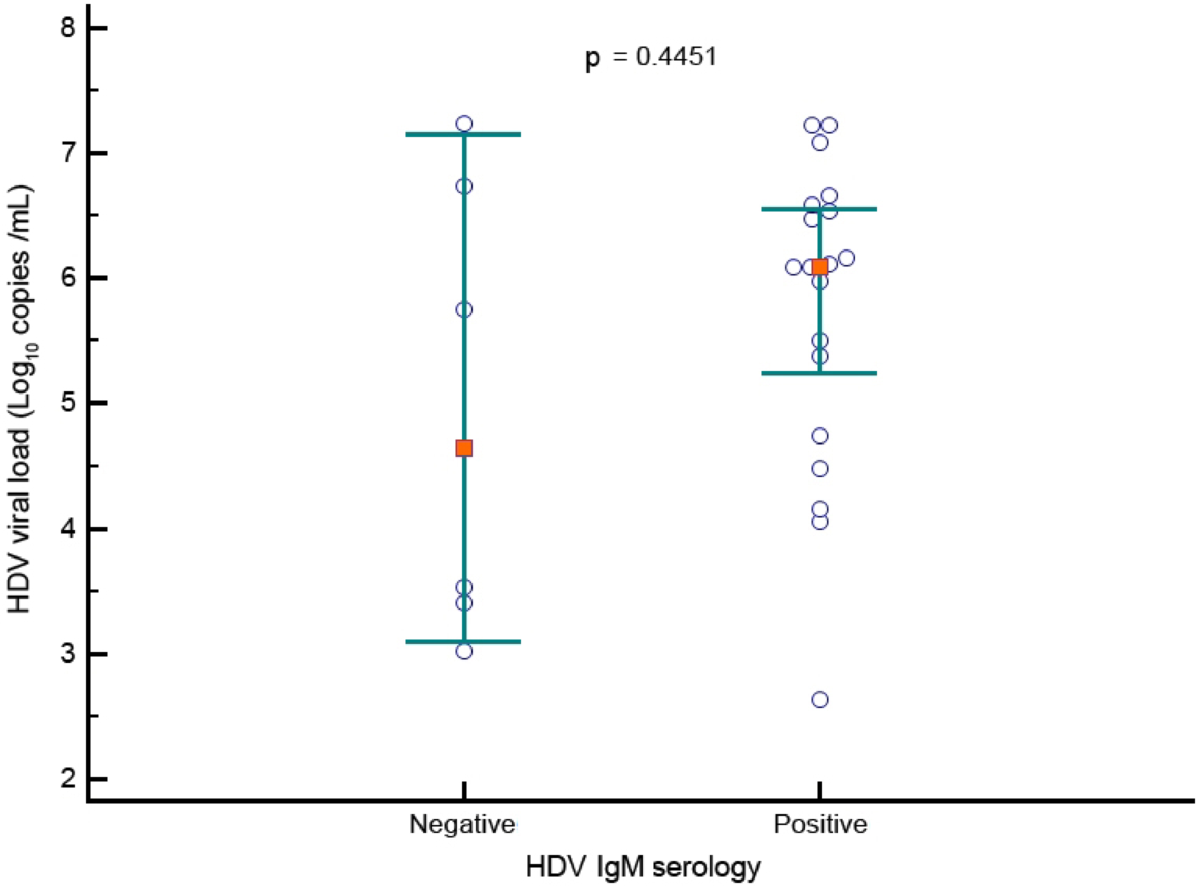

Technical Appendix Figure 2. HDV RNA log_{10} copies/mL in IgM-negative/HDV RNA positive (n=6) and IgM-positive/HDV RNA positive (n=19) samples. The orange square indicates the median and the error bars indicate 95% CI.
